# PROTOCOL: Community‐based interventions for initiating early end‐of‐life conversations in nonterminally Ill adults: a systematic review

**DOI:** 10.1002/cl2.1168

**Published:** 2021-05-24

**Authors:** Mervyn Jun Rui Lim, Qi Xuan Joel Foo, Noreen Guek Cheng Chan, Samuel Miny

**Affiliations:** ^1^ National University Health System (NUHS) Singapore Singapore; ^2^ Yong Loo Lin School of Medicine National University of Singapore Singapore Singapore; ^3^ Research Support Unit NUS Yong Loo Lin School of Medicine Singapore Singapore

## Abstract

This systematic review aims to answer the following research questions: (1) What are the existing community‐based interventions for initiating advance care planning (ACP) conversations and quality end‐of‐life (EoL) planning behaviours in nonterminally ill adults internationally? (2) What are the effects of community‐based interventions on the initiation of ACP conversations and EoL planning behaviours of nonterminally ill adults in the community?

## 1 BACKGROUND

### 1.1 Description of the condition

Quality end‐of‐life (EoL) care is emerging as an important public health issue globally. EoL care refers to care in the last 6–12 months of life (Goh, 2016). This is usually at the onset of a life‐limiting illness such as advanced cancer or end‐stage organ failure. EoL care aims to optimise quality of life through the provision of psychological, social, spiritual, and physical support for patients and their loved ones (DH, 2008). Insufficient EoL care conversations and preparation have resulted in unwanted admissions and treatments for patients with advanced diseases when it would not have altered their disease trajectory (Prince‐Paul & Difranco, 2017). With an ageing population and an increasing proportion of chronic illnesses with prolonged terminal phases (e.g., dementia), there is a pressing need to improve early EoL care preparation across the globe. For the purposes of this review, we define early EoL planning as planning for EoL care prior to the EoL (life expectancy of 12 months or less).

Early EoL planning has been shown to increase the quality of EoL care received (Brinkman‐Stoppelenburg et al., 2014), reduce admissions in the last 30 days of life (Prater et al., 2019), and increase quality of care near the EoL (Ankuda & Meier, 2018). Planning for EoL care requires conversations that are iterative and centre around the values of the person, encompassing topics such as care plans, medical plans, legal issues, and funeral planning (Sorrell, 2018). Currently, EoL conversations tend to be initiated by healthcare professionals, and usually only towards the last phase of a serious illness. As these tend to be medico‐centric, there is a tendency to neglect physical and psychosocial concerns (Balaban, 2000). Moreover, people with newly diagnosed life‐limiting illness often find it difficult to have EoL conversations at this time (Larson & Tobin, 2000).

Advance care planning (ACP) is a form of EoL communication process through which adults of any age or stage of health deliberate and share about their values, wishes and preferences regarding future medical care (Sudore et al., 2017). ACP involves EoL care preparation and has been shown to improve not only EoL care in patients but also psychological outcomes in family members (Detering et al., 2010). According to the Centers for Disease Control and Prevention, improving community education of ACP addresses the public health issue of unwanted and expensive treatments (Prince‐Paul & Difranco, 2017) and therefore, deliver quality EoL care. By having early ACP conversations and planning in the community before the last phase of a serious illness, family members and loved ones have sufficient time to explore EoL care as a wholistic concept that addresses not just the medical decisions, but also the social, legal and financial dimensions (Banner et al., 2019). Care plans that the family believe in and support may be made in advance, and can be instituted should the patient lose mental capacity due to illness.

Currently, early ACP conversations in the community are generally avoided by the public (especially in Asian societies), as ACP conversations may be considered taboo (Goh, 2018). A survey conducted in Singapore showed that only 36% of respondents from the general public expressed feeling comfortable having ACP conversations (Blackbox Research, 2014). Similar results have been reported in the UK with 72% of respondents agreeing with the statement “People in Britain are uncomfortable discussing dying, death, and bereavement” while they are healthy (ComRes, 2015), and in the United States, where 69% of respondents say that death is a subject that is generally avoided in their society (Kaiser Family Foundation, 2017).

Barriers to early ACP conversations include perceptions that ACP conversations are not relevant to individuals given their health or age (McLennan et al., 2015), concerns about relationships with loved ones and “burdening” them with such discussions (Schickedanz et al., 2009), lack of knowledge of EoL issues and options for EoL care (McCarthy et al., 2010), lack of accessibility to information for facilitating early ACP conversations (Banner et al., 2019), religious and cultural factors (Granek et al., 2013; Periyakoil et al., 2016), as well as a fear of dying (Tay et al., 2012).

While there exists multiple reports in the international literature focusing on community‐based interventions for initiating early ACP conversations and EoL planning behaviours, the effects of these interventions are poorly understood (Abba et al., 2013). Thus, in order to better understand the initiation of ACP conversations in nonterminally ill adults in the community, this review aims to summarise the existing international literature on community‐based interventions, and evaluate the effects of these interventions on the initiation of early ACP conversations and EoL planning in the community.

### 1.2 Description of the intervention

This review will include all types of community‐based interventions targeted at initiating early ACP conversations and EoL planning behaviours in nonterminally ill adults. For example, ACP conversations may include one or more of the following elements of early EoL care: reflection and discussion of personal values; preferences for medical care and/or treatment towards the EoL; preferences related to supportive, cultural, or spiritual care towards the EoL; concerns associated with personal finance and administrative documentation; concerns associated with the dying process; funeral planning; or death. Examples of EoL planning behaviours may include one or more of the following: completing an advanced directive document; making early funeral arrangements; making or updating a will; appointing a Lasting Power of Attorney (LPA). The type of intervention can include a range of approaches, including workshops, lectures, forums, roadshows, communication through brochures, or targeted one‐to‐one communication.

Community‐based interventions are complex interventions, which may be described as “non‐standard, having different forms in different contexts, while still conforming to specific, theory driven processes” (Petticrew, 2011). They have multiple interacting components and often do not have simple linear pathways linking the intervention and outcome. Thus, randomisation may not always be feasible and appropriate, and systematic reviews of complex health interventions will need to include data from a broader range of study designs (Shepperd et al., 2009).

An example of a randomised community‐based intervention for early EoL planning is the PREPARE randomised clinical trial (Sudore et al., 2018). In the PREPARE trial, older patients (55 years and above), with 2 or more chronic illnesses were recruited from primary care clinics in a public‐health delivery system. Participants were exposed to either both an easy‐to‐read version of an advance directive in addition to an online, patient‐directed ACP program (PREPARE arm); or just the advance directive alone. A subsequent increase in ACP documentation was reported in the PREPARE arm, compared to those exposed to the advance directive alone (43.0% vs. 33.1%, *p* < .001). Furthermore, there was higher patient‐reported engagement (98.1% vs. 89.5%, *p* < .001) in the PREPARE arm. Another randomised trial assessed the effect of a tripartite educational intervention (involving face‐to‐face discussion, educational forms and reminder cards) on the use and discussion of advanced directives (Sachs et al., 1992).

Examples of nonexperimental studies for early EoL community‐based interventions include lectures delivered by a physician to members of the general public who had responded to an open advertisement (Miyashita et al., 2008), roadshows held in busy town centres in collaboration with a local “Dying Matters” campaign aimed to educate and engage the public in discussing EoL issues (Hickey & Quinn, 2012), and the development of an information booklet and education programme for other elders by peer volunteers (Sanders et al., 2006).

### 1.3 How the intervention might work

These community‐based interventions aim to change the health behaviours of nonterminally ill adults in the community in order to nudge them towards early ACP conversations and EoL planning. As these community‐based interventions are complex behaviour‐change interventions that may impact multiple interacting components of a person's psychology and environment to change intention and behaviour, theoretical models on health behaviour change might provide us with greater insight on how these interventions might work. As these are a mixed‐bag of community‐based interventions, it is unlikely that a single theoretical model would be able to comprehensively explain the underpinnings of how such interventions could result in behaviour change in ACP conversations. However, it may be useful to understand different concepts of behaviour change theory, and how these may explain the effects of these community‐based interventions in increasing early ACP conversations and EoL planning behaviours in the community.

For example, the Health Belief Model (HBM) describes six concepts to health behaviour change: risk susceptibility, risk severity, benefits to action, barriers to action, self‐efficacy, and cues to action (Jones et al., 2015). The HBM proposes that individuals with greater perceived benefits, fewer perceived barriers, and good self‐efficacy who are provided cues to action are more likely to be motivated towards a health behaviour. In the context of EoL discussions, this is supported by a study which showed that the likelihood of EoL communication increased as perceived barriers decreased (Ko & Lee, 2009). For example, a public lecture series conducted in Japan addressed the misconception that dying at home was not possible. Prior to the lecture, 9% of participants stated that home death was possible. This increased to 34% after the lecture (Miyashita et al., 2008).

Another model on health behaviour change, the Theory of Planned Behaviour (TPB), described the concepts of an individual's attitudes, subjective norms, and perceived behavioural control on the intention to perform a particular health behaviour (Ajzen, 1985). The TPB was used in understanding healthy older adults’ intention to use hospice care should they face terminal illness (Nahapetyan et al., 2019). Nahapetyan et al. showed that higher perceived control to use hospice care, and preferences for EoL care that favour comfort and quality of life over living as long as possible were significant predictors of intention to use hospice care. For example, an education programme was developed for 121 older adults in West Japan. This included a video, a lecture with handouts, and a session for discussion among participants. By offering them an opportunity to learn more about EoL care and demystifying the dying process, improved attitudes towards advance directives (Matsui, 2010).

**Figure 1:**
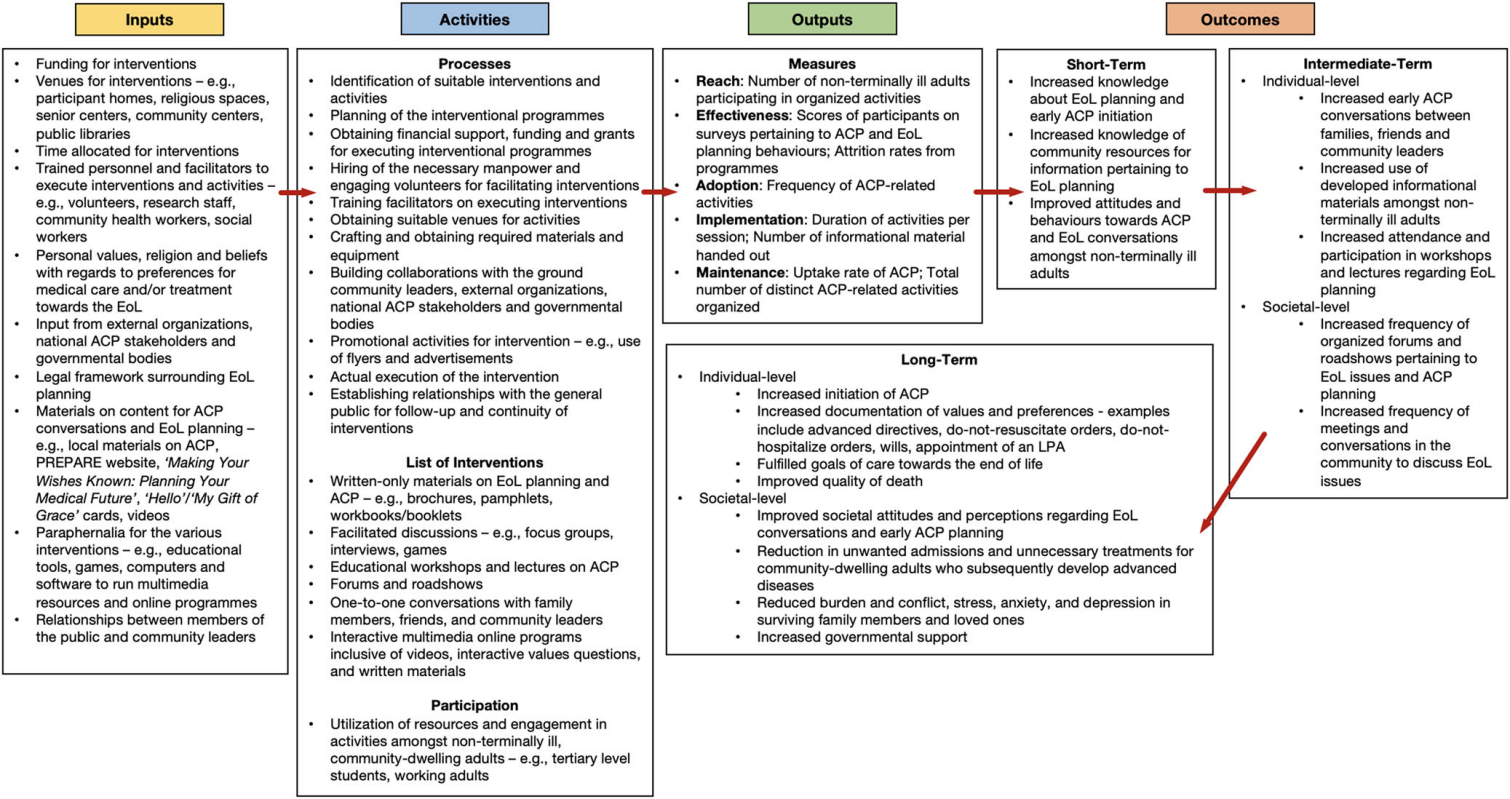
Logic model on the inputs, processes and outcomes of community‐based interventions for initiating advance care planning conversations in nonterminally Ill adults

Hence, by utilising theoretical models on health behaviour change such as the HBM and the TPB, we gain an insight into how these community‐based interventions can influence nonterminally ill adults to initiate early ACP conversations and adopt EoL planning in the community. In addition, we developed a logic model a priori (Figure 1) to better illustrate the inputs, processes, and outputs that in general, may be involved in such community‐based interventions, as well as how these may lead towards the short‐term and long‐term outcomes of the interventions (Bravo et al., 2016; Glasgow et al., 2019; Green & Levi, 2009; McMahan et al., 2021; RE‐AIM, 2021; Sudore et al., 2014; Van Scoy, Green, et al., 2017; Van Scoy, Reading, et al., 2017).

A limitation of these community‐based interventions are that these studies aim to increase outcomes of early ACP conversations and EoL planning in the community, but are unable to evaluate if these interventions change actual behaviour in future circumstances of approaching death or terminal illness. It is likely to be impractical and resource intensive to conduct such a study to evaluate if actual behaviour in the future has been impacted by the community‐based interventions that we are reviewing, and we acknowledge this as a limitation of the types of intervention that we are reviewing.

### 1.4 Why it is important to do this review

While there exists multiple reports in the international literature focusing on community‐based interventions for initiating early ACP conversations and EoL planning behaviours in nonterminally ill adults, the effects of these interventions are poorly understood. Furthermore, these community‐based interventions may be costly and resource intensive, or difficult to implement in other countries and cultural contexts. Hence, it is important to summarise the reported interventions and investigate their effects in the community.

To our knowledge, there is only one systematic review examining interventions to encourage discussion of EoL preferences between members of the general population and their loved ones (Abba et al., 2013). However, this review was conducted in 2013, included only five unique studies, and only examined the effects of discussions between participants and people close to them, or barriers to such a discussion. Thus, our systematic review will provide a comprehensive and up‐to‐date summary of complex community‐based interventions for the initiation of early ACP conversations and EoL planning behaviours that may aid other countries and societies in adopting such implementations.

## 2 OBJECTIVES

This systematic review aims to answer the following research questions:

1. What are the existing community‐based interventions for initiating ACP conversations and EoL planning behaviours in nonterminally ill adults internationally?
2. What are the effects of community‐based interventions on the initiation of ACP conversations and EoL planning behaviours of nonterminally ill adults in the community?

## 3 METHODS

### 3.1 Criteria for considering studies for this review

#### 3.1.1 Types of studies

We will include all experimental (e.g., randomised controlled trials), quasi‐experimental (e.g., pre‐post studies, nonrandomised trials, noncontrolled trials), and observational studies (e.g., cohort, case‐control, and cross‐sectional studies) that evaluate the effects of community‐based interventions for initiating early ACP conversations and EoL planning behaviours. Community‐based interventions are complex interventions and may be difficult to perform in a randomised or quasi‐randomised fashion. We expect to find a limited number of RCTs and quasi‐RCTs on this topic and therefore plan to include other study designs. Observational studies will also be included in order to provide an extensive overview of all interventions reported in this field.

We will exclude descriptive studies of an intervention (e.g., how an intervention was developed, or how an intervention was implemented, with no details on the effects of the intervention on early ACP conversations and EoL behaviour) and economic analyses of the interventions.

#### 3.1.2 Types of participants

This review will include nonterminally ill adults (aged 18 years old and above) in the community. Patients with chronic medical conditions may be included in the study as long as they do not fulfil the definition of terminally ill patients. Terminally ill patients are defined as patients with a life‐limiting illness and an expected life expectancy of 12 months or less. They may have the following trajectories of chronic illnesses (Lynn & Adamson, 2003):

1. Patients with a predictable progressive decline of health with a clear terminal phase. An example would be metastatic cancer; or
2. Patients with chronic life‐limiting conditions that have a gradual decline with interjecting episodes of acute decline and recovery, and the risk of sudden demise. Examples include end‐stage chronic obstructive pulmonary disease, cardiac failure, or renal failure.

Exclusion criteria: Studies conducted involving terminally ill participants or participants not in the community (inpatient or institutionalised participants) will be excluded from this review as they may already be motivated to initiate EoL planning, or may have already been exposed to ACP conversations through other platforms. Similarly, community‐based outreach to terminally ill adults residing in the community will be excluded from this study. Interventions targeting carers or family members of people with terminal illnesses within the context of the management of patients with terminal illnesses will also be excluded from this study.

As the focus of this review is on community‐based interventions, studies that were conducted on hospitalised participants or participants institutionalised in a healthcare facility will not be included in this review. This includes participants who are hospitalised, residing in nursing homes, mental institutions, community hospitals, inpatient hospice, or shelters and senior group homes. Individuals in the community may include those identified at an outpatient clinic, primary care, day care centres, senior care or activity centres.

#### 3.1.3 Types of interventions

All studies describing community‐based interventions for initiating early ACP conversations and EoL planning behaviours will be included. Interventions may include a range of approaches, including workshops, lectures, forums, roadshows, communication through brochures, or targeted one‐to‐one communication. These interventions may be conducted by government‐affiliated, private, or social organisations, or may be community‐based interventions that were initiated by members of the community. As these are complex interventions, a control group may not always be included in these interventions. The minimum requirements for an intervention to be included in this review are that it involves the topic of early ACP conversations and EoL planning in the community, and generates at least one outcome of early EoL planning behaviour.

##### Types of settings

All community‐based interventions will be included in this review. Individuals in the community may include participant homes, community centres, common spaces in the community, malls, schools, business compounds, religious spaces, and community healthcare facilities such as the outpatient clinic, primary care, day care centres, senior care or activity centres. As the focus of this review is on community‐based interventions, studies that were conducted on hospitalised participants or participants institutionalised in a healthcare facility will not be included in this review. This includes participants who are hospitalised, residing in nursing homes, mental institutions, community hospitals, inpatient hospice, or shelters and senior group homes.

#### 3.1.4 Types of outcome measures

We will include studies that evaluate the following primary or secondary outcomes using quantitative data (e.g., uptake rates, self‐reported scores on intention to initiate EOL planning behaviours, self‐reported scores on perceptions and attitudes to ACP). Studies that reported qualitative information (e.g., description of in‐depth interviews questions and methods of administering questions; focus group discussions; unstructured written narratives or responses from questionnaires) without quantitative data will be excluded from this study. In addition, outcomes evaluated by government‐affiliated, private, social organisations, or health institutions will be included in this study.

##### Primary outcomes

The primary outcomes of this review are the initiation of:

1. Early ACP conversations including discussions on one or more of the following elements of early EoL care: reflection and discussion of personal values; preferences for medical care and/or treatment towards the EoL; preferences related to supportive, cultural, or spiritual care towards the EoL; concerns associated with personal finance and administrative documentation; concerns associated with the dying process; funeral planning; or death; and
2. Early EoL planning behaviours including one or more of the following: completing an advanced directive document; making early funeral arrangements; making or updating a will; or appointing a LPA.

##### Secondary outcomes

Secondary outcomes include changes in knowledge, perceptions and attitudes towards early ACP conversations and early EOL planning behaviours.

###### Duration of follow‐up

No restrictions will be placed on the duration of follow‐up. In general, most community‐based interventions for early EoL planning may have a cross‐sectional or pre‐post design and may not have a follow‐up evaluation of the intervention as it may be difficult to recontact participants of such a community‐based intervention. However, some studies may include a follow‐up evaluation of the intervention at a later timeframe (e.g., 6 months postintervention) (Sato et al., 2009). This review will describe the presence of follow‐up and its respective duration if the included studies have explicitly stated so.

### 3.2 Search methods for identification of studies

#### 3.2.1 Electronic searches

We will search the following electronic databases from inception to the present time:

- Medline (via PubMed)
- Embase (via Embase.com)
- Cochrane Central Register of Controlled Trials (CENTRAL) in the Cochrane Library
- PsycINFO (OvidSP)
- Cumulative Index to Nursing and Allied Health Literature (CINAHL) (via EBSCO)

The search strategy will comprise of medical subject headings (MeSH) terms and keywords involving the concepts of (1) the outcome of the study includes early ACP conversations or early EoL planning, (2) types of interventions, and (3) studies conducted in the community. We have presented the electronic search strategy in Appendix A. We will apply no language nor date restrictions during our electronic search.

#### 3.2.2 Searching other resources

In order to further extend the search, the bibliographic references of relevant systematic reviews and all included articles will be searched for relevant articles to be included in the review. Relevant individuals and organisations will be contacted for information regarding unpublished or ongoing studies. We will also perform a search of the grey literature using Web of Science, Google Scholar, and the Worldwide Hospice Palliative Care Alliance.

### 3.3 Data collection and analysis

#### 3.3.1 Selection of studies

Two reviewers will independently screen the titles and abstracts of the retrieved records, to determine which meet the predefined inclusion criteria based on (a) the population of nonterminally ill adults in the community; and (b) interventions on early EoL planning behaviours using the Covidence tool (Veritas Health Innovation Covidence, 2020). Any discrepancies in records included will be resolved by discussion with a third review author.

The full texts of all included articles based on the title and abstract screen will be obtained online through institutional access. Where the full text articles are not readily available online, the authors will be contacted via email for the article. Articles that cannot be obtained even after contacting the original authors will be excluded from the study. Two reviewers will independently screen the full‐text articles for inclusion or exclusion and will resolve discrepancies by discussion and consultation with a third review author if necessary. We will list all potentially relevant papers excluded from the review at this stage as excluded studies and will provide reasons for exclusion in the “Characteristics of excluded studies” table. The screening and selection process will be reported according to the Preferred Reporting Items for Systematic Reviews and Meta‐Analyses (PRISMA) Statement (Moher et al., 2010).

#### 3.3.2 Data extraction and management

For studies that fulfil the inclusion criteria (randomised controlled trials, quasi‐experimental [e.g., pre‐post studies, nonrandomised trials, noncontrolled trials], and observational studies [e.g., cohort, case‐control, and cross‐sectional studies]), two review authors will independently conduct the data extraction using covidence with the following data fields: sample size, methodological issues (study design, sampling method, randomisation methods (if applicable), study setting, country, time frame, method used for data collection), demographic information (age, gender, race, education, income, marital status), interventions (type of intervention, platform of delivery, message of the intervention, time required to administer the intervention, frequency of administration of the intervention, follow up methods and time points of interest, comparison group if there is one, and cost of the intervention), and outcome data (initiation of early ACP conversations or EoL planning behaviours, changes in knowledge, perceptions and attitudes to early ACP conversations and EoL planning). Any discrepancies in data collection will be resolved by discussion with a third review author until a common consensus has been reached. We will contact investigators by email to request further data on methods or results where necessary.

#### 3.3.3 Assessment of risk of bias in included studies

Risk of bias assessment of selected studies will be conducted by two review authors independently using the National Institute for Health and Care Excellence (NICE) quality appraisal checklists for quantitative intervention studies and quantitative association studies (NICE 2012a, 2012b). Studies will be rated as “poor”, “fair”, or “good” quality based on these quality appraisal checklists. Discrepancies in the risk of bias assessment will be resolved by discussion with a third review author until a common consensus has been reached.

#### 3.3.4 Measures of treatment effect

For all studies with a control group, dichotomous data be analysed based on the number of events and the number of people assessed in the intervention and comparison groups. We will use these numbers to calculate the risk ratio (RR) or odds ratio (OR) and the 95% confidence interval (CI). In the instance that observational studies do not report number of events and the number of people assessed in the intervention, adjusted and unadjusted ORs and their respective 95% CIs will be extracted.

For continuous measures, we will analyse data based on the mean, the *SD*, and the number of people assessed for both intervention and comparison groups to calculate the mean difference (MD) and 95% CI. If more than one study measures the same outcome using different tools, we will calculate the standardised mean difference (SMD) and the 95% CI using the inverse variance method in Review Manager (RevMan) 2014. In cases of missing *SD*s, we will recalculate them from the reported statistics provided in these studies (e.g., CIs, standard errors, *p* values).

For quasi‐experimental studies where outcome measures are recorded pre‐ and postintervention, measures obtained before the intervention will be treated as the control group and measures obtained after the intervention as the treatment group (Reeves et al., 2020).

Further descriptive synthesis of various aspects of the community‐based interventions (e.g., the cost of the intervention, type of intervention, platform of delivery, message of the intervention, or time required for the intervention) will be extracted, if possible. Using the data collected, we will provide a comprehensive summary of all community‐based interventions for initiating early EoL planning and empirically categorise the range of interventions into broad groups based on how the intervention was conducted.

#### 3.3.5 Unit of analysis issues

For each included study, we will determine whether the unit of analysis is appropriate for the unit of randomisation and the design of each study (i.e., whether the number of observations matches the number of “units” that were randomised) (Higgins et al., 2020). The preferred method for unit of randomisation will be the individual participant.

If we include a cluster‐randomised trial, we will use the intraclass correlation coefficient to convert trials to their effective sample size before incorporating them into the meta‐analysis, as recommended by the Cochrane Handbook for Systematic Review of Interventions (Higgins et al., 2020).

Where insufficient data are available from cross‐over studies to incorporate paired data in a meta‐analysis, we will consider the measurements from each arm separately, as if from a parallel group trial. As this can result in a unit of analysis error, we will only include the results if they are demonstrably similar to the results of a paired analysis (Higgins et al., 2020).

Studies including within‐subject comparisons (pre‐ and post‐) will be included in the meta‐analysis if they analyse the data using paired sample *t* test and present the mean of the difference as well as its standard error. Then, such a result will be pooled with other studies presenting the MDs and standard errors using the generic variance approach in Review Manager.

#### 3.3.6 Dealing with missing data

In the event of any missing data, the review team will attempt to contact study authors via email to obtain the original data pertaining to the study. Where missing data cannot be recovered, methods used to cope with missing data will be used and any inherent assumptions will be reported, with the potential impact on findings discussed.

#### 3.3.7 Assessment of heterogeneity

If we judge that the included studies are too clinically heterogeneous to warrant a formal meta‐analysis, we will not perform a meta‐analysis but instead present the results of the included trials in a narrative format. We will assess clinical and methodological heterogeneity by comparing the distribution of important participant factors between studies, and study design factors, intervention types and loss to follow‐up.

We will explore statistical heterogeneity by visual inspection of the forest plots, using a standard *χ* ^2^ test, and a significance level of 0.1. We will also assess statistical heterogeneity in each meta‐analysis using the *I* ^2^ statistic described in the Cochrane Handbook for Systematic Reviews of Interventions recommendations (Higgins et al., 2020). We will identify heterogeneity using the following categories: <25%, no heterogeneity; 25%–40%, low heterogeneity; 50%–74%, moderate heterogeneity; and 75% or greater, high heterogeneity. We will explore substantial heterogeneity (≥75%), if identified, using subgroup analyses. We will explore clinical variation across studies by comparing the distribution of important participant factors in the study (e.g., age), and other study factors.

In addition to the *I* ^2^ value (Higgins et al., 2020), we will present the *χ* ^2^ statistic and its *p* value and consider the direction and magnitude of the treatment effects. As in meta‐analyses with few studies (Higgins et al., 2020), the *χ* ^2^ test is underpowered to detect heterogeneity should it exist; a *p* value of .10 will be used as a threshold of statistical significance.

#### 3.3.8 Assessment of reporting biases

To minimise the risk of publication bias, we will attempt to obtain the results of any unpublished studies in order to compare findings extracted from published reports with results from other sources. If there are more than 10 studies grouped in a comparison, we will assess whether reporting biases are present using funnel plots to investigate any relationship between effect estimates and study size/precision, as recommended in the Cochrane Handbook for Systematic Reviews of Interventions Version 6.1 (Higgins et al., 2020). An Egger's test will be performed for asymmetrical funnel plots. We will attempt to distinguish potential causes of reporting biases should there be an asymmetrical funnel plot, and any potential impact on the findings will be discussed.

#### 3.3.9 Data synthesis

Results for randomised trials, quasi‐experimental designs, and observational studies will be synthesised and reported separately. One review author will enter all extracted data into RevMan (Review Manager 5.4), and a second review author working independently will check the data for accuracy against the extracted data.

We will decide whether to meta‐analyse data based on whether interventions in the included trials are similar enough in terms of participants, settings, interventions, comparisons, and outcome measures to ensure meaningful conclusions from a statistically pooled result.

If two or more studies have adequate data, we will conduct a meta‐analysis of the available data for each outcome (e.g., uptake rate of ACP completion). We anticipate variability in populations and interventions, setting, and possibly other factors, of included studies. Therefore, we will combine RRs/ORs or SMDs using a random effects model and a forest plot will be reported.

If there are more than 10 studies, contour‐enhanced plots will be constructed and statistical tests of funnel plot asymmetry will be performed (Egger's and Harbord's test). Heterogeneity will be assessed using both Cochran's *Q* test and the *I* ^2^ statistic (Higgins et al., 2020).

If we are unable to pool the data statistically using meta‐analysis, we will report data using descriptive statistics and narrative synthesis, or thematic synthesis *n* order to provide readers with a broad overview on the effects of these community‐based interventions on the design elements of the interventions, change in knowledge and attitudes of early EoL planning behaviours in the community, and other effects of these interventions.

#### 3.3.10 Subgroup analysis and investigation of heterogeneity

Subgroup analyses will be performed in order to assess if there is variation in the effects of the intervention depending on the following factors:

1. Study design (e.g., randomised trials, quasi‐experimental, and observational studies).
2. Type of intervention and design elements of the intervention (platform of delivery, frequency of intervention).
3. Country or region where the study was conducted.
4. Population demographics (age group (≥50 and ≤50), gender, education level).

#### 3.3.11 Sensitivity analysis

In the event that there is a sufficient number of studies identified describing the same interventions, sensitivity analysis will be conducted for the primary outcomes to determine whether the review conclusions would have differed if the following factors were considered:

1. Study eligibility restricted to studies with only fair‐to‐good quality.
2. Study setting restricted to studies that did not involve healthcare settings in the community.

#### 3.3.12 Summary of findings and assessment of the certainty of the evidence

We will prepare a “Summary of findings” table to present results for the main outcomes, based on the methods described in Chapter 11 of the Cochrane Handbook for Systematic Reviews of Interventions (Schünemann et al., 2011).

The outcomes will include:

##### 3.3.12.1 Primary outcomes

1. Initiation of early ACP conversations by the participants including discussions on one or more of the following elements of early EoL care: reflection and discussion of personal values; preferences for medical care and/or treatment towards the EoL; preferences related to supportive, cultural, or spiritual care towards the EoL; concerns associated with personal finance and administrative documentation; concerns associated with the dying process; funeral planning; or death; and
2. Early EoL planning behaviours including one or more of the following: completing an advanced directive document; making early funeral arrangements; making or updating a will; or appointing a LPA.

##### 3.3.12.2 Secondary outcomes

Changes in knowledge, perceptions and attitudes towards early ACP conversations and early EOL planning behaviours.

We will prepare a “Summary of findings” table for the following comparisons:

- Community‐based interventions (Interventions may include a range of approaches, including workshops, lectures, forums, roadshows, communication through brochures, or targeted one‐to‐one communication) for initiating early ACP conversations and EoL planning behaviours versus control or no intervention.
  We will present the results for each of the major primary outcomes as outlined in the types of outcome measures section for comparison. We will provide a source and rationale for each assumed risk cited in the table(s), and we will use the GRADE system to rank the certainty of evidence using GRADEprofiler (GRADEpro) software. Two review authors will independently rate the certainty of each outcome, and any disagreements will be resolved via discussion. We will report the certainty of evidence according to GRADE using the five criteria: study limitations (risk of bias), inconsistency, indirectness, imprecision, and publication bias.
  We will define the levels of evidence as “high”, “moderate”, “low”, or “very low”. These grades are defined as follows.
- High certainty: this research provides a very good indication of the likely effect; the likelihood that the effect will be substantially different is low.
- Moderate certainty: this research provides a good indication of the likely effect; the likelihood that the effect will be substantially different is moderate.
- Low certainty: this research provides some indication of the likely effect; however, the likelihood that it will be substantially different is high.
- Very low certainty: this research does not provide a reliable indication of the likely effect; the likelihood that the effect will be substantially different is very high.

## CONTRIBUTIONS OF AUTHORS

The roles and responsibilities of the authors are as follows:

- *Content*: Mervyn Jun Rui Lim and Noreen Guek Cheng Chan.
- *Systematic review methods*: Mervyn Jun Rui Lim and Samuel Miny.
- *Statistical analysis*: Mervyn Jun Rui Lim and Samuel Miny.
- *Information retrieval*: Mervyn Jun Rui Lim and Qi Xuan Joel Foo.

## SOURCES OF SUPPORT

### Internal sources

- NUS Yong Loo Lin School of Medicine, Singapore.
- National University Health Systems, Singapore.

### External sources

Campbell Social Welfare Group, Norway.

## APPENDIX A SEARCH STRATEGY

### MedLine (via PubMed) (retrieved 8977 studies)

**Table:**

No	Search Query	Results
1	"Terminal Care"[MeSH Terms] OR "Hospice Care"[MeSH Terms] OR "Advance Care Planning"[MeSH Terms] OR "Advance Directives"[MeSH Terms] OR "Attitude to Death"[MeSH Terms] OR "Terminal Care*"[Title/Abstract] OR "end‐of‐life"[Title/Abstract] OR "end of life"[Title/Abstract] OR "hospice care*"[Title/Abstract] OR "euthanasia"[Title/Abstract] OR "resuscitation order*"[Title/Abstract] OR "palliative care*"[Title/Abstract] OR "advance care*"[Title/Abstract] OR "advanced care*"[Title/Abstract] OR "advance directive*"[Title/Abstract] OR "advanced directive*"[Title/Abstract] OR ((Attitude[Title/Abstract] OR attitudes[Title/Abstract]) AND (death*[Title/Abstract] OR die[Title/Abstract] OR dies[Title/Abstract] OR dying[Title/Abstract])) OR living will* [Title/Abstract] OR Power of Attorney[Title/Abstract] OR funeral plan*[Title/Abstract])	113346
2	("Health Promotion"[MeSH Terms] OR "Mass Media"[MeSH Terms] OR "Health Education"[MeSH Terms] OR "Communication"[MeSH Terms] OR "Focus Groups"[MeSH Terms] OR "Decision Making"[Mesh] OR "Motivational Interviewing"[Mesh] OR "Self‐Help Groups"[Mesh] OR "Health Promotion"[Title/Abstract] OR "education*"[Title/Abstract] OR "workshop*"[Title/Abstract] OR "training*"[Title/Abstract] OR "program"[Title/Abstract] OR "programs"[Title/Abstract] OR "programme"[Title/Abstract] OR "programmes"[Title/Abstract] OR "Talk"[Title/Abstract] OR "Talks"[Title/Abstract] OR discussion*[Title/Abstract] OR "brochure*"[Title/Abstract] OR "public forum"[Title/Abstract] OR "Mass Media"[Title/Abstract] OR "intervention*"[Title/Abstract] OR "focus group*"[Title/Abstract] OR "presentation*"[Title/Abstract] OR "road show"[Title/Abstract] OR "road shows"[Title/Abstract] OR conversation*[Title/Abstract] OR plan*[Title/Abstract] OR Motivational Interview*[Title/Abstract] OR “peer mentoring”[Title/Abstract] OR “self‐help group*”[Title/Abstract] OR “support group*”[Title/Abstract])	4657493
3	("Community Health Planning"[MeSH Terms] OR "Senior Centers"[MeSH Terms] OR "Adult Day Care Centers"[MeSH Terms] OR "Community Participation"[MeSH Terms] OR "Community"[Title/Abstract] OR "Communities"[Title/Abstract] OR "neighbourhood"[Title/Abstract] OR "day care"[Title/Abstract] OR "home care"[Title/Abstract] OR "senior centre*"[Title/Abstract] OR "senior center*"[Title/Abstract] OR "primary care"[Title/Abstract] OR “outpatient clinic*”[Title/Abstract] OR school*[Title/Abstract] OR church[Title/Abstract] OR churches[Title/Abstract] OR temple[Title/Abstract] OR temples[Title/Abstract] OR mosque[Title/Abstract] OR mosques[Title/Abstract] OR “religious centre”[Title/Abstract] OR “religious center”[Title/Abstract] OR “place of worship”[Title/Abstract] OR “places of worship”[Title/Abstract])	1069088
4	((("Terminal Care"[MeSH Terms] OR "Hospice Care"[MeSH Terms] OR "Advance Care Planning"[MeSH Terms] OR "Advance Directives"[MeSH Terms] OR "Attitude to Death"[MeSH Terms] OR "Terminal Care*"[Title/Abstract] OR "end‐of‐life"[Title/Abstract] OR "end of life"[Title/Abstract] OR "hospice care*"[Title/Abstract] OR "euthanasia"[Title/Abstract] OR "resuscitation order*"[Title/Abstract] OR "palliative care*"[Title/Abstract] OR "advance care*"[Title/Abstract] OR "advanced care*"[Title/Abstract] OR "advance directive*"[Title/Abstract] OR "advanced directive*"[Title/Abstract] OR ((Attitude[Title/Abstract] OR attitudes[Title/Abstract]) AND (death*[Title/Abstract] OR die[Title/Abstract] OR dies[Title/Abstract] OR dying[Title/Abstract])) OR living will* [Title/Abstract] OR Power of Attorney[Title/Abstract] OR funeral plan*[Title/Abstract])) AND (("Health Promotion"[MeSH Terms] OR "Mass Media"[MeSH Terms] OR "Health Education"[MeSH Terms] OR "Communication"[MeSH Terms] OR "Focus Groups"[MeSH Terms] OR "Decision Making"[Mesh] OR "Motivational Interviewing"[Mesh] OR "Self‐Help Groups"[Mesh] OR "Health Promotion"[Title/Abstract] OR "education*"[Title/Abstract] OR "workshop*"[Title/Abstract] OR "training*"[Title/Abstract] OR "program"[Title/Abstract] OR "programs"[Title/Abstract] OR "programme"[Title/Abstract] OR "programmes"[Title/Abstract] OR "Talk"[Title/Abstract] OR "Talks"[Title/Abstract] OR discussion*[Title/Abstract] OR "brochure*"[Title/Abstract] OR "public forum"[Title/Abstract] OR "Mass Media"[Title/Abstract] OR "intervention*"[Title/Abstract] OR "focus group*"[Title/Abstract] OR "presentation*"[Title/Abstract] OR "road show"[Title/Abstract] OR "road shows"[Title/Abstract] OR conversation*[Title/Abstract] OR plan*[Title/Abstract] OR Motivational Interview*[Title/Abstract] OR "peer mentoring"[Title/Abstract] OR "self‐help group*"[Title/Abstract] OR "support group*"[Title/Abstract]))) AND (("Community Health Planning"[MeSH Terms] OR "Senior Centers"[MeSH Terms] OR "Adult Day Care Centers"[MeSH Terms] OR "Community Participation"[MeSH Terms] OR "Community"[Title/Abstract] OR "Communities"[Title/Abstract] OR "neighbourhood"[Title/Abstract] OR "day care"[Title/Abstract] OR "home care"[Title/Abstract] OR "senior centre*"[Title/Abstract] OR "senior center*"[Title/Abstract] OR "primary care"[Title/Abstract] OR "outpatient clinic*"[Title/Abstract] OR school*[Title/Abstract] OR church[Title/Abstract] OR churches[Title/Abstract] OR temple[Title/Abstract] OR temples[Title/Abstract] OR mosque[Title/Abstract] OR mosques[Title/Abstract] OR "religious centre"[Title/Abstract] OR "religious center"[Title/Abstract] OR "place of worship"[Title/Abstract] OR "places of worship"[Title/Abstract]))	8977

### Embase (via Embase.com) (retrieved 7095 studies)

**Table:**

No	Search Query	Results
1	'terminal care'/exp OR 'hospice care'/exp OR 'advanced care planning'/exp OR 'advanced directives' OR 'attitude to death'/exp	80613
2	'terminal care*':ti,ab OR 'end‐of‐life':ti,ab OR 'end of life':ti,ab OR 'hospice care*':ti,ab OR euthanasia:ti,ab OR 'resuscitation order*':ti,ab OR 'palliative care*':ti,ab OR 'advance care*':ti,ab OR 'advanced care*':ti,ab OR 'advance directive*':ti,ab OR 'advanced directive*':ti,ab OR ((attitude:ti,ab OR attitudes:ti,ab) AND (death*:ti,ab OR die:ti,ab OR dies:ti,ab OR dying:ti,ab)) OR 'living will*':ti,ab OR 'power of attorney':ti,ab OR 'funeral plan*':ti,ab	103640
3	'health promotion'/exp OR 'mass communication'/exp OR 'health education'/exp OR 'interpersonal communication'/exp OR 'decision making'/exp OR 'motivational interviewing'/exp OR 'self help'/exp	1843910
4	('health promotion':ti,ab OR 'education*':ti,ab OR 'workshop*':ti,ab OR 'training*':ti,ab OR 'program':ti,ab OR 'programs':ti,ab OR 'programme':ti,ab OR 'programmes':ti,ab OR 'talk':ti,ab OR 'talks':ti,ab OR discussion*:ti,ab OR 'brochure*':ti,ab OR 'public forum':ti,ab OR 'mass media':ti,ab OR 'intervention*':ti,ab OR 'focus group*':ti,ab OR 'presentation*':ti,ab OR 'road show':ti,ab OR 'road shows':ti,ab OR conversation*:ti,ab OR plan*:ti,ab OR motivational:ti,ab) AND interview*:ti,ab OR 'peer mentoring':ti,ab OR 'self‐help group*':ti,ab OR 'support group*':ti,ab	251655
5	'health care planning'/exp OR 'senior center'/exp OR 'adult day care'/exp OR 'community participation'/exp	107615
6	'community':ti,ab OR 'communities':ti,ab OR 'neighbourhood':ti,ab OR 'day care':ti,ab OR 'home care':ti,ab OR 'senior centre*':ti,ab OR 'senior center*':ti,ab OR 'primary care':ti,ab OR 'outpatient clinic*':ti,ab OR school*:ti,ab OR church:ti,ab OR churches:ti,ab OR temple:ti,ab OR temples:ti,ab OR mosque:ti,ab OR mosques:ti,ab OR 'religious centre':ti,ab OR 'religious center':ti,ab OR 'place of worship':ti,ab OR 'places of worship':ti,ab	1302295
7	(#1 OR #2) AND (#3 OR #4) AND (#5 OR #6)	7095

### The Cochrane Library (retrieved 802 studies)

**Table:**

No	Search Query	Results
1	MeSH descriptor: [Terminal Care] explode all trees	462
2	MeSH descriptor: [Advance Care Planning] explode all trees	234
3	MeSH descriptor: [Attitude to Death] explode all trees	154
4	("Terminal Care*" OR "end‐of‐life" OR "end of life" OR "hospice care*" OR euthanasia OR "resuscitation order*" OR "palliative care*" OR "advance care*" OR "advanced care*" OR "advance directive*" OR "advanced directive*" OR ((Attitude OR attitudes) AND (death* OR die OR dies OR dying)) OR "living will*" OR "funeral plan*" OR "Power of Attorney"):ti,ab	4276
5	#1 OR #2 OR #3 OR #4	4531
6	MeSH descriptor: [Health Promotion] explode all trees	6556
7	MeSH descriptor: [Mass Media] explode all trees	1862
8	MeSH descriptor: [Health Education] in all MeSH products	20248
9	MeSH descriptor: [Communication] explode all trees	8557
10	MeSH descriptor: [Focus Groups] explode all trees	627
11	MeSH descriptor: [Decision Making] explode all trees	3988
12	MeSH descriptor: [Motivational Interviewing] explode all trees	865
13	MeSH descriptor: [Self‐Help Groups] explode all trees	775
14	("Health Promotion" OR education* OR workshop* OR training* OR program OR programs OR programme OR programmes OR Talk OR Talks OR discussion* OR brochure* OR "public forum" OR "Mass Media" OR intervention* OR "focus group*" OR presentation* OR "road show" OR "road shows" OR conversation* OR plan* OR "Motivational Interview*" OR "peer mentoring" OR "self‐help group*" OR "support group*"):ti,ab	569152
15	#6 OR #7 OR #8 OR #9 OR #10 OR #11 OR #12 OR #13 OR #14	576392
16	MeSH descriptor: [Community Health Planning] explode all trees	48
17	MeSH descriptor: [Senior Centers] explode all trees	19
18	MeSH descriptor: [Adult Day Care Centers] explode all trees	13
19	MeSH descriptor: [Community Participation] explode all trees	1686
20	("Community" OR "Communities" OR "neighbourhood" OR "day care" OR "home care" OR "senior centre*" OR "senior center*" OR "primary care" OR “outpatient clinic*” OR school* OR church OR churches OR temple OR temples OR mosque OR mosques OR “religious centre” OR “religious center” OR “place of worship” OR “places of worship”):ti,ab	91666
21	#16 OR #17 OR #18 OR #19 OR #20	92819
22	#5 AND #15 AND #21	802

### CINAHL (via EBSCO) (retrieved 4544 studies)

**Table:**

No	Search Query	ResultsHeading
S1	(MH "Terminal Care"+) OR (MH "Advance Care Planning") OR (MH “Advance Directives”+) OR (MH “Attitude to Death”+)	3835
S2	TI (("Terminal Care*" OR "end‐of‐life" OR "end of life" OR "hospice care*" OR euthanasia OR “resuscitation order*" OR "palliative care*" OR "advance care*" OR "advanced care*" OR "advance directive*" OR "advanced directive*" OR ((Attitude OR attitudes) AND (death* OR die OR dies OR dying)) OR “living will*” OR “Power of Attorney” OR “funeral plan*”)) OR AB (("Terminal Care*" OR "end‐of‐life" OR "end of life" OR "hospice care*" OR euthanasia OR “resuscitation order*" OR "palliative care*" OR "advance care*" OR "advanced care*" OR "advance directive*" OR "advanced directive*" OR ((Attitude OR attitudes) AND (death* OR die OR dies OR dying)) OR “living will*” OR “Power of Attorney” OR “funeral plan*”))	53208
S3	(MH "Health Promotion"+) OR (MH "Mass Media"+) OR (MH “Communication”+) OR (MH “Focus Groups”) OR (MH "Decision Making + ") OR (MH "Support Groups + ") OR (MH "Motivational Interviewing")	188136
S4	TI ("Health Promotion" OR "education*” OR "workshop*" OR "training*" OR "program" OR "programs" OR "programme" OR "programmes" OR "Talk" OR "Talks" OR discussion* OR "brochure*" OR "public forum" OR "Mass Media" OR "intervention*" OR "focus group*" OR "presentation*" OR "road show" OR "road shows" OR conversation* OR plan* OR Motivational Interview* OR “peer mentoring” OR “self‐help group*” OR “support group*”) OR AB ("Health Promotion" OR "education*” OR "workshop*" OR "training*" OR "program" OR "programs" OR "programme" OR "programmes" OR "Talk" OR "Talks" OR discussion* OR "brochure*" OR "public forum" OR "Mass Media" OR "intervention*" OR "focus group*" OR "presentation*" OR "road show" OR "road shows" OR conversation* OR plan* OR Motivational Interview* OR “peer mentoring” OR “self‐help group*” OR “support group*”)	1437137
S5	(MH "Community Health center"+) OR (MH "Adult Day Care Centers") OR (MH "Community Networks") OR (MH "Community Role")	6682
S6	TI ("Community" OR "Communities" OR "neighbourhood" OR "day care" OR "home care" OR "senior centre*" OR "senior center*" OR "primary care" OR “outpatient clinic*” OR school* OR church OR churches OR temple OR temples OR mosque OR mosques OR “religious centre” OR “religious center” OR “place of worship” OR “places of worship”) OR AB ("Community" OR "Communities" OR "neighbourhood" OR "day care" OR "home care" OR "senior centre*" OR "senior center*" OR "primary care" OR “outpatient clinic*” OR school* OR church OR churches OR temple OR temples OR mosque OR mosques OR “religious centre” OR “religious center” OR “place of worship” OR “places of worship”)	483266
S7	(S1 OR S2) AND (S3 OR S4) AND (S5 OR S6)	4544
